# Balancing beliefs: exploring preservice teachers’ generative artificial intelligence perceptions and intentions for practice

**DOI:** 10.3389/frai.2026.1834667

**Published:** 2026-06-01

**Authors:** Mike Karlin, Cristina Stephany, 'Alohilani Okamura, Yoon Jin Nam-Huh

**Affiliations:** 1 Liberal Studies, California State University, Dominguez Hills, Carson, CA, United States; 2 Palos Verdes Peninsula Unified School District, Palos Verdes Estates, CA, United States; 3 School of Teacher Education, University of Hawai'i at Manoa, Honolulu, HI, United States

**Keywords:** case study, generative artificial intelligence, K-12 education, preservice teachers, teacher education

## Abstract

Generative artificial intelligence (GenAI) tools such as ChatGPT have rapidly entered educational contexts, raising questions about their affordances, limitations, and implications for teacher preparation. This qualitative, exploratory case study investigated preservice teachers’ perceptions of ChatGPT across two sites in Los Angeles and Hawai‘i, with 74 participants. Data were collected through surveys embedded in interactive learning modules and analyzed using thematic analysis. Findings indicated alignment in perceived affordances for both students and teachers, including idea generation, and the importance of knowing how to use tools properly. Participants also emphasized limitations such as risks of dependency, plagiarism, and exposure to bias or misinformation. While most preservice teachers expressed willingness to use ChatGPT in their future teaching, few supported future student use. Across responses, participants highlighted the need for training to ensure ethical and effective implementation. This study underscores the importance of integrating both technical and critical AI literacy into teacher education.

## Introduction

Though generative artificial intelligence (GenAI) has only been available to the public since 2022, it has already had a significant impact on the field of education (Kasneci et al., 2023; Tlili et al., 2023). The initial reaction to GenAI tools (e.g., ChatGPT) by many school districts, including Los Angeles Unified School District (the second largest district in the United States) was to ban the tool and block access (Mearian, 2023). Teacher education programs and institutes of higher education in general have also engaged in conversations and policy discussions related to GenAI use (McMurtrie, 2023). Discussions have ranged from urging faculty and universities to rethink coursework (Scott, 2023), redesign assessments (Lo, 2023), and potentially ban GenAI tools altogether (Mearian, 2023). To add to these conversations, continuing questions and concerns exist related to the reliability, accuracy, and equity-related consequences of GenAI detectors used to identify and flag AI-generated content. (Weber-Wulff et al., 2023). Finally, considerations around the environmental impacts of GenAI (e.g., Kshetri, 2024) as well as the spread of misinformation within GenAI tools have also emerged as significant concerns (e.g., Chu-Ke and Dong, 2024).

In general, foundational GenAI knowledge that includes basic tool use, how models are constructed, bias in datasets, and the ethical and social impacts of AI are important for all students to learn to be both critical users and creators with technology (Wong et al., 2020). Since Large Language Models (LLMs) are increasingly being adopted as foundational tools within social institutions such as healthcare, government, and education, users require knowledge and skills to be able to utilize tools responsibly as well as recognize and mitigate hallucinations within outputs. Hallucinations are inaccuracies in responses generated by AI models that appear to be authoritative, yet are rooted in inherent model biases or inaccurate/incomplete training artifacts (Anh-Hoang et al., 2025). For example, our future depends on medical doctors who can decipher whether the AI models they use have racist or sexist biases, as well as legislators and community members who understand AI deeply enough to identify misinformation, ethically construct new laws about technology-use, and oversee cases about technology-related issues.

This foundational education begins in K-12 schools and is not possible without highly qualified and well-trained preservice teachers (Ottenbreit-Leftwich et al., 2023). As GenAI continues to make rapid advancements that impact numerous aspects of our daily lives, teachers and students need to be prepared with the knowledge and skills to understand and address these advancements (Touretzky et al., 2019), as well as the myriad of ways AI impacts and informs our interactions with the world around us (Wong et al., 2020). However, for students to begin to develop this necessary AI literacy, teachers must also be prepared to teach and integrate AI-related concepts into their curricula (Ottenbreit-Leftwich et al., 2023). As the uptake of AI continues in schools, limited emerging studies exist that attempt to understand the potential impacts of GenAI tools for education, particularly for preservice teachers within teacher education pathways (e.g., Bae et al., 2024).

Broadly, this study focuses on preservice teachers’ perceptions of the GenAI LLM ChatGPT as a tool for teaching and learning. ChatGPT is a GenAI LLM developed by the company OpenAI, and since its initial release in 2022, approximately 10% of the world’s adult population has adopted ChatGPT, with 18 billion weekly messages being sent across 700 million users (Chatterji et al., 2025). Given ChatGPT’s expansive and growing influence in the GenAI space, alongside a growing number of higher education and ChatGPT partnerships (e.g., California State University, Office of the Chancellor, 2025), this tool was particularly relevant for exploring current trends in GenAI within the broad context of higher education and the specific context of teacher education pathways.

More specifically, there is a dearth of research that examines preservice teachers’ perceptions of the affordances and limitations of ChatGPT. Gibson developed the concept of “affordances” in his 1979 book, The Ecological Approach to Visual Perception. Gibson’s Theory of Affordances (1979) refers to the relationship between surfaces in an environment (objects) and animals or people (actors). Affordances are what the environment offers, provides, or furnishes to the actor, and Gibson’s work explores how values and meanings related to these affordances can be directly perceived. Relying on Gibson’s (1979) work on affordances, this current study also builds on modern relevant studies such as Butler and Jiang’s (2025) research that explores the relationship between preservice teachers and ChatGPT to understand their perceptions of affordances and limitations.

### Research questions

Given the expanding and influential presence of GenAI tools like ChatGPT within K-12 and preservice education, alongside the important role preservice teachers play in the adoption and implementation of these tools, this study sought to build upon the understanding of how preservice teachers perceive GenAI tools like ChatGPT as tools for teaching and learning within their future classrooms by asking the following questions:

(1) What do preservice teachers report as potential affordances and limitations of ChatGPT for *students*?
(2) What do preservice teachers report as potential affordances and limitations of ChatGPT for *teachers*?
(3) How (if at all) do preservice teachers plan to use GenAI tools like ChatGPT in their future classroom?

## Literature review

The need to understand teachers’ perceptions of ChatGPT is essential to developing teacher training, and the need to develop teacher training is apparent in emerging studies that examine students’ perceived affordances of ChatGPT. For example, in a mixed-methods study utilizing the Student Attitudes Toward Artificial Intelligence scale and the Artificial Intelligence Anxiety Scale, Sivenas (2025) examined 109 Greek high school students’ interactions with ChatGPT-5, focusing on their attitudes, anxiety, and responses to hallucinations. Additionally, semi-structured interviews were conducted with 36 of the students. The findings included the following student-reported pedagogical affordances: Generating new knowledge; immediate feedback; friendly and familiar user interface; skill development; uncertainty about content accuracy; anxiety related to AI feedback; and privacy and data protection concerns. Overall, the findings suggest that the educational potential of ChatGPT is not only shaped by model advancements, but also by the extent to which educators can develop curricula that guide students to engage with these tools critically and mitigate issues such as hallucinations (Sivenas, 2025). Thus, preservice teachers must understand the pedagogical implications of GenAI tools not only for themselves as teachers, but also for the students they serve (Bae et al., 2024).

Yet, studies have shown that there are low adoption rates of AI tools among preservice teachers. Kalnina et al. (2024) surveyed 240 preservice teachers at the University of Latvia to examine AI usage patterns and perceived benefits and challenges. Overall, the results showed that less than half of preservice teachers within the sample use AI tools in their learning. Additionally, less than half were positive about AI tools and almost a third thought that AI tools should be banned in the study process. Also concerning was that almost a quarter did not know if they were using AI or not. The study highlighted the low awareness of AI in education at this university and emphasized the need for preservice teachers to acquire AI literacy, such as prompt formulation and practical AI application, as well as ethical and responsible use of tools. The results also included preservice teachers’ perceptions of the opportunities and challenges of using AI in higher education. Preservice teachers identified overcoming language barriers, making global knowledge more accessible, and helping to implement inclusive principles (aiding those with disabilities) as opportunities. Identified challenges related to ethical aspects of AI, such as unfair use, false information, and the reduction of the learning process. The authors suggest further research is needed to investigate preservice teachers’ beliefs about AI in the learning and teaching process, which will inform how universities prepare future teachers to realize the full potential of AI in education and mitigate risks (Kalnina et al., 2024).

Additionally, studies have analyzed preservice teachers’ perceptions of GenAI tools beyond survey results. Bae et al. (2024) collected and analyzed discussion posts from preservice teachers enrolled in asynchronous online courses focused on ChatGPT. The authors found that preservice teachers tended to envision using GenAI tools primarily for lesson planning and brainstorming teaching ideas, rather than for the full spectrum of instruction. Preservice teachers also expressed concern that over-reliance on GenAI might undermine students’ critical thinking, creativity, and ethical reasoning.

In another study, Yang and Appleget (2024) first generated questions with GenAI during a read-aloud activity with elementary preservice teachers, then used a qualitative survey to collect data about how the activity influenced their preservice teachers’ perceptions of GenAI. The data analysis was grounded within the Technology Acceptance Model (TAM) framework to understand the participants’ acceptance or rejection of GenAI. Of the 56 preservice teacher participants, 77% of which had none or little prior experience with GenAI before the day of the activity, 91% viewed GenAI as a useful tool for teaching. Additionally, about half the participants who supported GenAI also had concerns about the use for educational purposes. The preservice teachers valued creativity, agency, accountability, and critical reasoning while using GenAI and perceived it to be a collaborator rather than a replacement teacher (Yang and Appleget, 2024). The authors call for teacher educators’ collaborative exploration of GenAI with their preservice teachers as a pathway for access, experience, and understanding of GenAI tools.

Lastly, studies have focused on understanding preservice teachers’ perceptions of the affordances of ChatGPT. Butler and Jiang (2025) examined 38 University of Pennsylvania preservice Teaching English to Speakers of Other Languages (TESOL) teachers’ perceptions of GenAI based on comparing evaluations of their own writing and writing generated by ChatGPT on the same topic. The findings showed that in comparative evaluation of the essays, the frequencies of participants’ positive and negative statements about ChatGPT were almost equal. Yet, participants who rated ChatGPT higher than their own writing had overconfidence in GenAI, while participants who rated their own writing higher were more critical of ChatGPT’s limitations. Butler and Jiang (2025) concluded that developing accurate knowledge of AI’s affordances and promoting balanced views of AI through professional development is not easy but necessary to make ethical and meaningful use of GenAI in teacher education.

**Figure 1:**
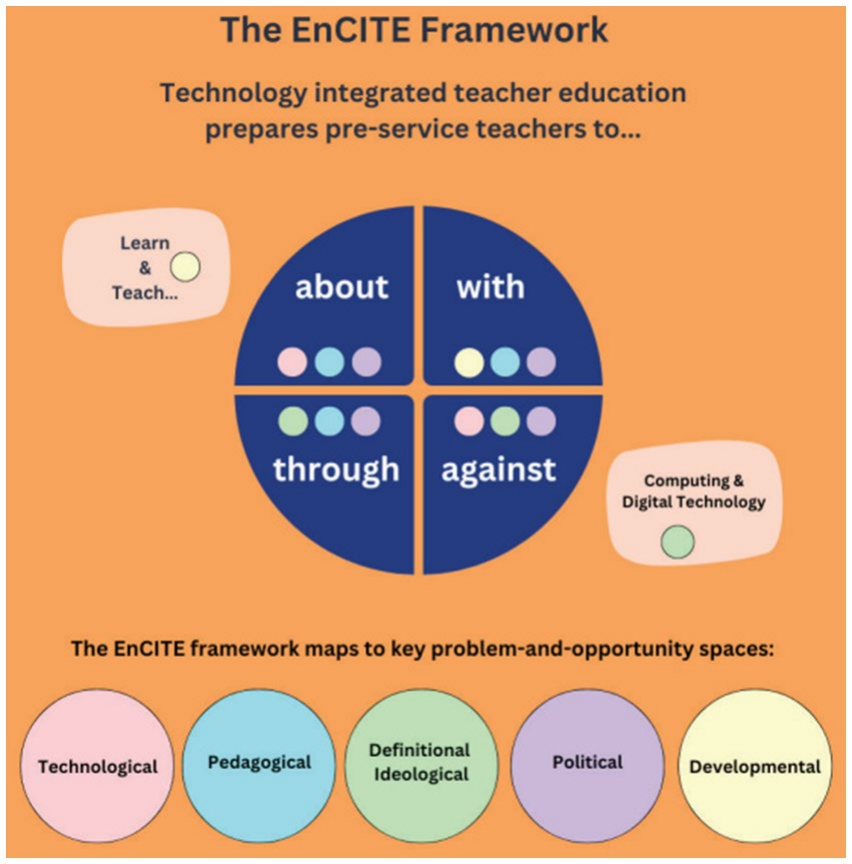
Vogel et al. (2024) EnCITE framework for preservice teachers, used with permission of author.

The “EnCITE” framework was developed specifically to grapple with these types of complex problems and opportunities afforded by integrating computing and digital technology into teacher education (Vogel et al., 2024). Researchers have argued that teacher education programs often teach and integrate education technology tools with the assumption that these technology tools are neutral and benign (Krutka et al., 2022, p. 229). However, when tools like ChatGPT are classified as neutral and integrated into teacher education programs alongside other technologies, these tools have the potential to be harmful, particularly to our students and communities who are most often underserved and marginalized (Benjamin, 2019; Noble, 2018). Vogel et al. (2024) proposed the EnCITE framework for use in teacher education where preservice teachers learn about, with, through, and against topics of computing and digital technologies (see Figure 1). This framework makes intentional space for the foundational learning of skills required for emerging technologies, but also for critical approaches that interrogate how technology might be used for unjust or problematic implementations. This study leverages the framework for the exploration of preservice teacher affordances of and future practices with GenAI.

The EnCITE framework identifies five interconnected problem-and-opportunity spaces, including the technological, pedagogical, definitional/ideological, political, and developmental spaces. For example, the political space focuses on inequities and how technology can perpetuate injustices. Anchoring preservice teachers’ perceptions of the affordances and limitations of ChatGPT within the EnCITE framework attends to the need to develop more equity-minded future educators.

Overall, the present study builds upon the research described above to understand preservice teachers’ perceptions of the affordances and limitations of ChatGPT and provides a culminating analysis of those perceptions from the lens of the EnCITE framework in the discussion section. Building upon the methods, findings, and questions posed from previous relevant research, the present study included a set of interactive modules to build standardized interactions between the preservice teacher participants and ChatGPT. Overall, this study moves forward existing work and findings around GenAI affordances in teacher education and contributes new contexts, experiences, voices, and recommendations to the discussion.

## Methods

This work presents results from a qualitative, exploratory case study that was conducted within the bounded contexts of Los Angeles and the islands of Hawai‘i (Yin, 2017). The unit of analysis was the “participant experience” described below. We recruited 40 preservice teacher participants from California State University, Dominguez Hills and 35 from the University of Hawai’i at Manoa. One student did not complete the study, leaving a total of 74 preservice teacher participants across two sites. This work was part of a larger study, as well as part of a terminated grant originally supported by the National Endowment for the Humanities.

### Context

As a Hispanic Serving Institution and a Minority Serving Institution, California State University, Dominguez Hills undergraduate programs consist of 78.8% of students from underrepresented groups (African American/Black, Latiné/Hispanic, and Native American), and 47.5% first-generation college students. University of Hawai’i at Manoa serves 63.5% underserved populations, including Native Hawaiian, Pacific Islander, and Asian students.

### Participant demographics

For the 74 participants in this study, 100% were preservice teachers, all at various stages of their program, but all with previous classroom and field experience. IRB approval was received for this study, and participants received a $200 stipend for their participation. Participant demographics are shown below in Table 1. In addition to English, a wide variety of languages were spoken by participants, including Spanish, Japanese, Ilokano, Tagalog, French, Samoan, and Russian.

**Table 1:** Participant demographics (*N =* 74).

Category	*n*	%
Race/Ethnicity		
Hispanic or Latine	25	34%
Asian or Asian American	19	26%
White	12	16%
Native Hawaiian/Pacific Islander	7	10%
Black or African American	6	8%
Mixed Race	4	5%
Prefer not to say	1	1%
Gender		
Female	57	77%
Male	15	20%
Non-binary	1	1%
Prefer not to say	1	1%
Age range		
18–20	6	8%
21–25	33	45%
26–30	13	18%
31–35	6	8%
36–40	8	11%
41–45	5	7%
46–50	2	3%
50+	1	1%

Table 2 shows future education direction and prior education experience, and Table 3 shows prior ChatGPT experience. Overall, Table 2 shows the diverse range of participant grade levels and future subject areas. Additionally, the majority of participants (*n =* 71, 96%) had at least some prior experience working in schools in a formal capacity (e.g., para educator, teacher assistant, etc.).

**Table 2:** Future teaching area, subject specializations, and prior experience (*N =* 74).

Category	*n*
Future teaching area	
Early Elementary (PreK–2)	36
Upper Elementary	40
Middle School	15
High School	20
Special Education	9
Future subject area (secondary)	
English	10
Japanese	3
Social Studies	2
Math	5
Physics	1
Biology	1
General Science	2
Spanish	3
Physical Education/Health	3
Art	1
Employed experience in education (e.g., para educator)	
None	3
Less than 1 year	11
1–3 years	30
3–5 years	14
5 + years	16

Participants could select multiple future teaching areas and subjects.

**Table 3:** Prior use of ChatGPT and reported use cases (*N =* 74).

Category	*n*	%
Previously used ChatGPT		
Yes	51	69%
Research/Homework	11	–
Lesson Planning	11	–
Brainstorming	10	–
Proofreading/Feedback	8	–
Professional Emails	7	–
Play/Exploration	6	–
Misc. Personal Use (e.g., travel, recipes)	6	–
Translations	2	–
No	23	31%
Not Exposed/No Access	9	–
Not Allowed/Nervous	5	–
Didn’t Need It	7	–
Worried About Quality	2	–

Participants could select multiple rationales.

For Table 3, participant background with ChatGPT is reported. The majority of participants (*n =* 51, 69%) had previously used ChatGPT, with the top three use cases being research and homework, lesson planning, and brainstorming. For those who had not previously used ChatGPT, the primary reason was due to either not having exposure or not having access. Comparisons between those with previous ChatGPT experience and those without are also examined in the results section below.

### Participant experience

**Figure 2:**
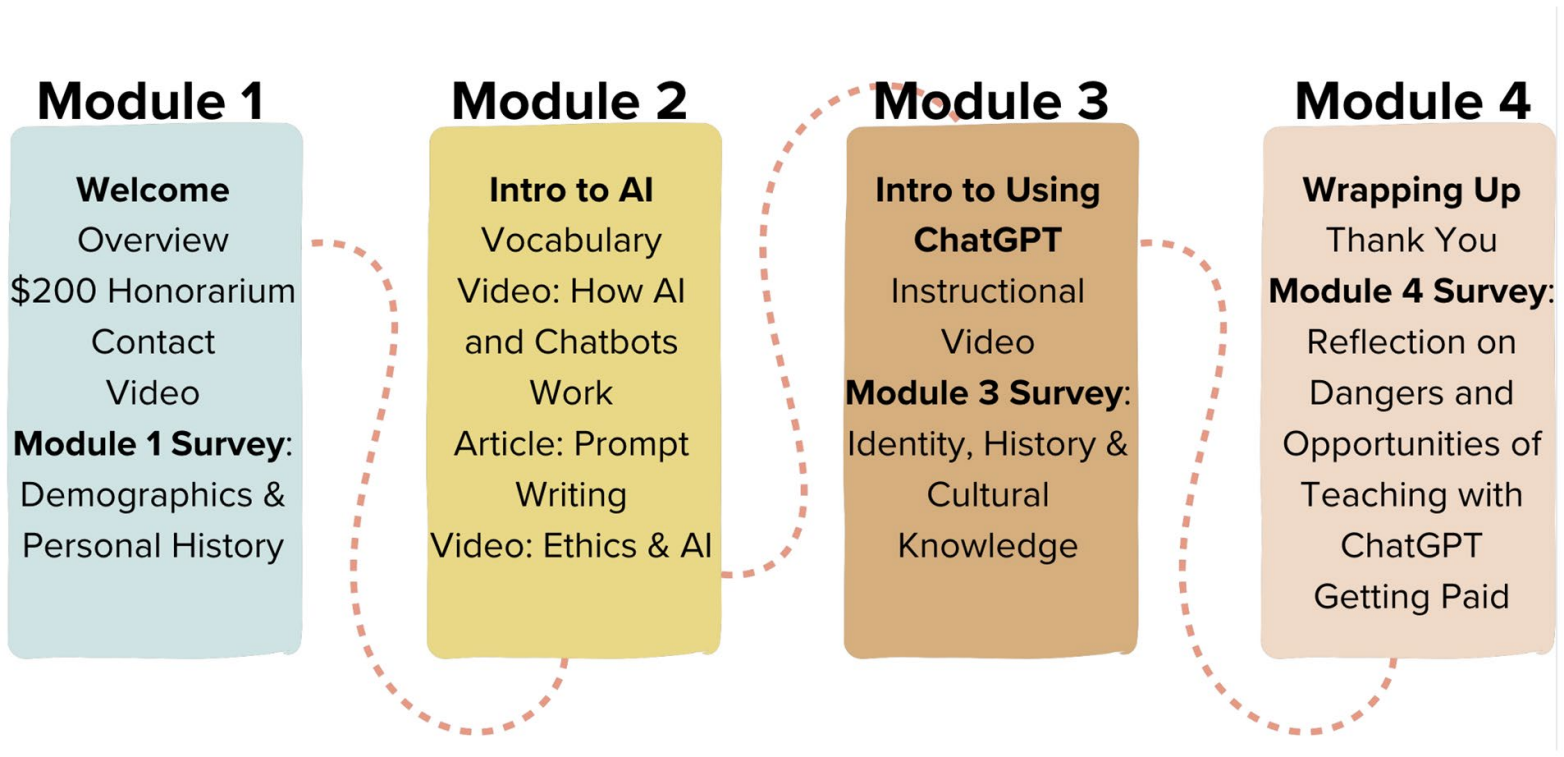
Structure and content of participant learning experience.

Participants completed interactive modules, consisting of: 1) Sharing demographic data and personal history; 2) learning about prompt generation and AI’s impact on society; 3) engaging with ChatGPT version 3.5 through a series of prompts, inserting their identity, history, and cultural knowledge and reflecting upon the responses; and 4) reflections on the dangers and opportunities of teaching with ChatGPT. Data were collected through surveys embedded within modules 1, 3, and 4. Modules were sequenced in a way to ground participants in foundational GenAI knowledge and skills, and topics included GenAI vocabulary, GenAI principles, prompt writing, ethics, and more (see Figure 2). Participants engaged with modules online, asynchronously, over a three-month time period during the summer.

### Data sources

Data collection occurred during the aforementioned participant experience, through surveys embedded within modules one, three, and four. Qualtrics was used as the survey collection instrument. Demographic and participant background information was collected in module one. In module two, a second survey was used as a practice survey, so that participants had an opportunity to practice the process of prompting ChatGPT and pasting their prompts and ChatGPT’s responses into the Qualtrics survey. Participants copied and pasted responses into Qualtrics (rather than uploading chat logs) to protect participant anonymity. Data from this practice survey were not used for analysis. As mentioned above, this work was part of a larger research study, which explored ChatGPT as a cultural tool, and tensions that might arise between identity characteristics presented by ChatGPT and individual self-perceptions. The specific prompts used for the larger study are presented below in Appendix A. For this study, data collected during module four were relied on for analysis (see Appendix B).

### Data analysis

To analyze the data, we utilized thematic analysis (Braun and Clarke, 2006) to identify the affordances and limitations perceived by participants. To begin, data from the three surveys were consolidated into one Excel file, and the lead researcher conducted the initial analysis of all survey data to determine initial codes. A bottom-up or inductive approach was utilized to uncover themes emergent in the data (Braun and Clarke, 2006). During this initial analysis by the lead researcher, primary, low-level codes were first developed, which contained minimal abstraction (Braun and Clarke, 2006).

Three members of the research team then met to review these initial codes. The three researchers looked for larger, abstracted trends across the low-level codes (Braun and Clarke, 2006), and the codes were consolidated and reduced to three final codebooks, aligned with each of the study’s three research questions (see Appendix A). Each response could be coded for multiple codes. For example, for the question “What are the dangers and opportunities of teachers using ChatGPT for educational purposes?” a participant’s reply of “Dangers is that students won’t learn and fully comprehend the topic. The opportunities it provides is that it helps speed up brainstorming and providing quick answers to simple questions.” was coded as both “Teacher Limitations Dependent” and “Teacher Affordance Exploration.”

After initial coding was completed and the final codebook established, the lead researcher then again re-coded all data to align with the finalized codes. Finally, rather than rely on inter-rater reliability, and to increase trustworthiness, all three team members met again to review and finalize all codes. In cases of disagreement on codes, discussion was held and three members of the research team engaged in arbitration until agreement was reached (Saldaña, 2015). Results from across the surveys that are aligned with the study’s three research questions are presented below.

### Limitations

The limitations of this study primarily relate to the self-reported survey response data, which can potentially hold a self-representation bias (Kopcha and Sullivan, 2007). In addition to self-representation bias, case studies often have limited generalizability. We have attempted to mitigate this limitation through the inclusion of multiple sites, and the inclusion of in-depth and complete participant survey responses. Finally, to maintain participant anonymity, we asked for copied and pasted ChatGPT responses in the survey as noted above, which allowed space for editing ChatGPT responses in survey submission. We have attempted to mitigate this limitation by focusing our analysis on participant experiences with ChatGPT engagement, rather than specific ChatGPT output.

## Results

The emergent themes from analysis are organized by research questions and explored in depth below. Additionally, analysis was conducted to explore differences between those with prior GenAI/ChatGPT usage and those without, which is reported within each section. Analysis was also conducted to explore differences between the two sites of the study (Los Angeles and Hawai’i) and no differences emerged for this portion of the study related to the themes reported on below. For accessibility, Table 4 below shows the overall results across RQ1 (Affordances and Limitations for Students) and RQ2 (Affordances and Limitations for Teachers).

**Table 4:** Summary of overall results across RQ1 (perceived affordances and limitations for students) and RQ2 (perceived affordances and limitations for teachers).

Perceived affordances			
Perceived affordances for students (RQ1)	Number/Percent of respondents	Perceived affordances for teachers (RQ2)	Number/Percent of respondents
Idea Generation	*n =* 43, 58%	Idea Generation	*n =* 31, 42%
Need Knowledge for Effective Use	*n =* 14, 19%	Lesson Planning	*n =* 27, 37%
Editing and Proofing	*n =* 8, 11%	Need Knowledge for Effective Use	*n =* 13, 18%

Perceived limitations			
Perceived limitations for students (RQ1)	Number/Percent of respondents	Perceived limitations for teachers (RQ2)	Number/Percent of respondents
Become Dependent	*n =* 35, 47%	Bias and Misinformation	*n =* 38, 51%
Plagiarism	*n =* 33, 45%	Become Dependent	*n =* 33, 45%
Bias and Misinformation	*n =* 29, 39%	Plagiarism (and Job Risks)	*n =* 7, 10%

### Research question 1: Affordances and limitations for students

In their survey responses, preservice teacher participants explored both affordances and limitations for their future students. Primary emergent themes for ChatGPT *affordances* for future students included: (1) Students may use ChatGPT for brainstorming and idea generation (*n =* 43, 58%); (2) Students need knowledge to use GenAI tools properly (*n =* 14, 19%); and (3) students may use ChatGPT for editing and proofing (*n =* 8, 11%). These themes are explored further below. Importantly, 20 participants reported *no* affordances for student use. These 20 participants instead focused only on the perceived limitations they had for student use of tools like ChatGPT.

Overall, the primary emergent themes for ChatGPT *limitations* for future students included: (1) Students may become dependent on ChatGPT (*n =* 35, 47%); (2) students may use ChatGPT for plagiarism (*n =* 33, 45%); and (3) students may receive misinformation from ChatGPT (*n =* 29, 39%).

#### Affordance one: idea generation

The primary emergent theme around affordances of GenAI tools like ChatGPT for students that preservice teachers reported on was using the tool for brainstorming and idea generation (*n =* 43, 58%). There was a wide range of ideas for what brainstorming and idea generation could look like for students, including supporting student research projects, but overall, over half of the participants mentioned ideas related to this theme. For example, one stated: “There are countless opportunities for students using ChatGPT. It can help students in so many ways, help come up with ideas and so much more.” Another noted:

ChatGPT can make research and learning so much faster for students who have questions or need answers to a more specific subject. ChatGPT saves time and quickly pulls information from multiple databases and feeds us the information into a concise and specific answer.

One respondent shared their optimism about the tool with a note of caution, “ChatGPT allows people to learn things quickly and opens up new information, but just as with any other tool, the value is in how it is used.” Finally, one participant simply remarked, “You can use [ChatGPT] as an inspiration.”

#### Affordance two: students need knowledge for effective use

The secondary emergent theme around affordances of GenAI tools like ChatGPT for students that preservice teachers reported on was that in order to have actual affordances, students need knowledge to use GenAI tools properly (*n =* 14, 19%). In other words, participants clarified that while affordances do exist, students must have training and instruction on how to use these tools properly and critically. For example, one participant noted that students “need to be educated that using AI may not always be accurate. I think it would be very helpful for ChatGPT to post under its response the sources so that they can be cross-checked.” Another responded, noting the importance of clear and specific prompts, saying ChatGPT “can provide very tailored explanations, especially the more specific you prompt it.” Finally, one respondent simplified this perception stating, “ChatGPT can be a very useful tool for those who know how and when to use it.”

#### Affordance three: editing and proofing

Finally, the tertiary emergent theme around the affordances of GenAI tools like ChatGPT for students that preservice teachers reported on was using the tool for editing and proofing (*n =* 8, 11%). For example, one respondent noted, “ChatGPT would be a great resource for students to use as a reference or to double-check certain facts or grammatical and punctuation errors.” Another said students “can use it as a resource to check their work.” Finally, a third mentioned how it could be beneficial for students based on their own experiences:

ChatGPT serves as a tool to students…who need help writing, and/or learning new information. ChatGPT is very valuable to me personally, since I am not the best writer. It is a tool that I use to help me reword sentences that I quickly write down and have trouble rephrasing to sound a little more organized and academic.

Importantly, for RQ1, this theme is the only point of difference between participants with prior GenAI/ChatGPT experience and those with no prior experience. Here, of the eight students who discussed this theme in their survey responses, seven of them had prior experience, and only one of the respondents had no prior experience.

#### Limitation one: students may become dependent

The primary emergent theme around the limitations of GenAI tools like ChatGPT for students that preservice teachers reported on was that students may become dependent on the tool (*n =* 35, 47%). For example, one participant stated: “Students can become dependent on ChatGPT, which reduces critical thinking and risks crossing the lines of student dishonesty. Students can overlook the learning process just by trying to get the right answers.” Another noted how students “can become overly reliant on this tool, which can hinder our independent thinking and academic integrity.” Finally, one participant replied, noting the social consequences of students becoming too dependent: “If they rely solely on [ChatGPT], they may not become critical thinkers. They may be easily manipulated by others.”

#### Limitation two: students may use ChatGPT to plagiarize

The secondary emergent theme around the limitations of GenAI tools like ChatGPT for students that preservice teachers reported on was that students may use the tool to plagiarize (*n =* 33, 45%). For example, a participant responded that one of their concerns was “students using ChatGPT to complete assignments for them instead of using it for support.” Another emphasized: “CHEATING! That is the most important thing that comes into my head when I think of ChatGPT. I think it could be used in order to get an idea of what could be done but students could cheat through it if they do not use it right.” Finally, one participant reported, “Students might present ChatGPT-generated work as their own. It can be misused for completing assignments or exams dishonestly.”

#### Limitation three: students may receive misinformation

Finally, the tertiary emergent theme around the limitations of GenAI tools like ChatGPT for students that preservice teachers reported on was that students may receive biased information or misinformation in general (*n =* 29, 39%). For example, one participant discussed the potential danger for their future students being exposed to misinformation: “I think the biggest danger is students being exposed to the ‘hallucinations’ and biases when working with ChatGPT. I would never want my students, especially students that are already vulnerable, to be exposed to that type of content.” Another echoed and simplified this sentiment, sharing that “ChatGPT is still very flawed from its inaccurate information to its prejudiced interpretations.” A third connected this theme to their own experiences in the research study, while also comparing ChatGPT to other tools, reporting:

Some dangers [of ChatGPT] are biased and inaccurate information. I have noticed through this survey and my own experiences that ChatGPT is kind of like a student who is trying to reach a word count instead of trying to find the most accurate information. However, this is true for most online resources. Many sources are not entirely trustworthy and/or biased.

Finally, one participant described the importance of integrating these ideas of bias and misinformation into pedagogical practices, sharing how:

Students may not recognize that ChatGPT has inherent biases, misinformation, or can promote certain misconceptions. It is important to relay to students these shortcomings and emphasize the fact that it is merely a tool for learning and not a substitute for education.

### Research question 2: Affordances and limitations for teachers

In their survey responses, preservice teacher participants explored both affordances and limitations for themselves as future teachers. Primary emergent themes for ChatGPT *affordances* for future teachers included: (1) Teachers may use ChatGPT for brainstorming and idea generation (*n =* 31, 42%); (2) teachers may use ChatGPT for lesson planning (*n =* 27, 37%); and (3) teachers need knowledge to use properly (*n =* 13, 18%); These themes are explored further below. Importantly, 14 (19%) respondents reported *no* affordances for teacher use, and no thematic differences were found between those with and without prior ChatGPT experience.

Primary emergent themes for ChatGPT *limitations* for teachers included: (1) Teachers may receive misinformation from ChatGPT (*n =* 38, 51%); (2) teachers may become dependent on ChatGPT (*n =* 33, 45%); and (3) Teachers may use ChatGPT for plagiarism or other job risks (*n =* 7, 10%).

#### Teacher affordance one: idea generation

The primary emergent theme around the affordances of GenAI tools like ChatGPT for teachers was using the tool for brainstorming and idea generation (*n =* 31, 42%). For example, one preservice teacher wrote how ChatGPT can support brainstorming when teachers “encounter and access ideas that they would not have thought of, and also ChatGPT can help save them time.” Another shared excitement around the possibilities that ChatGPT offered, writing, “The opportunities it has are incredible, they provided me with a lot of new ideas.” A third simply stated, “I find it a great tool for brainstorming ideas.”

#### Teacher affordance two: lesson planning

The secondary emergent theme around the affordances of GenAI tools like ChatGPT for teachers was using the tool for lesson planning (*n =* 27, 37%). For example, one preservice teacher wrote that “Teachers using ChatGPT can benefit from its ability to generate lesson plans, suggest teaching strategies, and provide resources quickly, enhancing their efficiency and creativity in the classroom.” Another suggested how using ChatGPT can make their lessons more engaging and relevant by using the tool to “keep their lessons modern, appeal and relate to their students [of any] age range.” Finally, a third wrote how “Teachers are able to use ChatGPT to get lesson ideas and outline of lesson plans.”

#### Teacher affordance three: teachers need knowledge for effective use

The tertiary emergent theme around the affordances of GenAI tools like ChatGPT for teachers was that (similar to students), affordances may not be realized unless teachers know how to use the tools properly and critically (*n =* 13, 18%). For example, one preservice teacher wrote, “I think teachers could get a lot out of [ChatGPT], but will probably need to spend some time getting a full understanding of its different functions and limitations.” Another noted what might happen if teachers do not have a full understanding of the tool, writing, “I fear that some teachers might use ChatGPT to help them develop lesson plans without knowing the importance of writing detailed and informative prompts.” Finally, one preservice teacher approached this theme from a philosophical standpoint, noting how:

Teachers may experience a disconnect with the human aspect of the occupation, one that drives a learning community. The human aspect of carefully crafting a curriculum that is accessible to all students will need to remain paramount to educators using ChatGPT.

#### Teacher limitation one: teachers may receive misinformation

The primary emergent theme around the limitations of GenAI tools like ChatGPT for teachers was that the tool may provide biased information or misinformation in general (*n =* 38, 51%). For example, one participant wrote that ChatGPT “does not incorporate the fact that all students are different and they all have their own personalities/needs when making lessons or plans in the classroom.” Similarly, another preservice teacher described how ChatGPT “may not account for the perspectives of students that are marginalized unless teachers are more informed on the use of AI immediately, and the ethical dangers.” Another respondent connected this theme to the danger of plagiarism as well, noting, “I fear that some teachers might just copy and paste lessons without reading them through or analyzing them for ‘hallucinations’ and other biases/ mistakes.” Finally, one preservice teacher connected this theme to the possibility of ChatGPT providing problematic or ineffective pedagogical strategies for students, writing:

ChatGPT occasionally provides incorrect, outdated, or overly simplified information, which could lead to the dissemination of inaccuracies in lessons or materials. Also, AI-generated content might lack the depth, nuance, or pedagogical strategies needed for effective teaching. It is CRUCIAL that teachers are formally trained with the background knowledge and skills to truly design and deliver lessons in a way that suits their students’ unique needs and interests.

#### Teacher limitation two: teachers may become dependent

The secondary emergent theme around the limitation of GenAI tools like ChatGPT for teachers was that they may become dependent on the tool (*n =* 33, 45%). For example, one respondent noted that if teachers become too reliant on ChatGPT, then “teachers [will not be] studying and learning material for themselves.” Another explored this sentiment further, writing that “the clear and present danger is that teachers will look up lesson plan ideas and copy and paste them without altering them in the slightest for their students’ individual context and cultural background.” Finally, one respondent made connections back to the perceived limitations for student use, explaining that “The dangers could be similar with students too, like being too dependent on ChatGPT.”

#### Teacher limitation three: teachers may use ChatGPT to risk job

The tertiary emergent theme around the limitations of GenAI tools like ChatGPT for teachers was that they may use the tool to plagiarize or that the use of the tool might lead to other job risks (*n =* 7, 10%). For example, one respondent wrote simply that using ChatGPT could lead to teachers “Violating copyright.” Outside of copyright and plagiarism concerns, many of these job risk concerns centered around data privacy concerns and the sharing of student information. One respondent wrote that “the main danger, however, is the data privacy and security concerns. At the moment of writing, there is no defined set of guidelines for teachers on data privacy regulations when using AI.” Another echoed this sentiment, writing: “The dangers of teachers using ChatGPT is that some can potentially include student information which needs to always be confidential.” Finally, a third respondent bluntly shared how these types of privacy concerns might lead to teachers losing their jobs: “A teacher using ChatGPT for educational purposes could have information passed on to administration who may want to talk. It may cost the teacher their job.”

### Research question 3: Future plans

Finally, preservice teacher participants reported on their plans for use of ChatGPT in their future classrooms, both for themselves as teachers and for their future students. Overall, 67 (91%) answered “Yes” or “Maybe” for planning to use ChatGPT as a teacher in their future classroom, and 14 (19%) answered “Yes” or “Maybe” for allowing or supporting their future students’ use. This sizable difference between future teacher use and future student use was primarily attributed to concerns about ChatGPT doing the work for students, and students missing out on skill and knowledge development as a result. While one participant reported they would not let students use ChatGPT because they did not trust the tool, all other participants who said they would not let students use ChatGPT expressed concerns like “it will lower students’ thinking process” and “I want to hear what my students think and if they can expand their thoughts without the use of AI.”

In terms of teacher use, the overall themes for research question three on how (if at all) teachers planned to use ChatGPT in their future classroom included: (1) Idea exploration and generation (*n =* 41, 55%); (2) lesson planning (*n =* 32, 43%); (3) need for more training and knowledge (*n =* 22, 30%). All three are explored further below.

#### Future use one: idea generation

The primary emergent theme around future use of GenAI tools like ChatGPT for teachers was for idea exploration and generation (*n =* 41, 55%). For example, one participant reported that ChatGPT can be helpful when facing writer’s block or trying to come up with an idea: “I could see myself using [ChatGPT], especially when it comes to getting an idea of what to write about when I am stuck. I believe there are times we may need assistance due to being stuck, but ChatGPT could help us out in this.” Another participant noted that ChatGPT can help generate ideas for increasing engagement in the classroom through humor or new problems, saying that ChatGPT “is an excellent tool for refining the teaching craft. For example, it can provide a time saver for creating problems, introducing humor into lessons, and, overall, increasing engagement in the classroom.” Overall, participant responses around plans for using ChatGPT for idea generation aligned with previously reported responses around perceived affordances for teachers and students.

#### Future use two: lesson planning

The secondary emergent theme around future use of GenAI tools like ChatGPT for teachers was for lesson planning (*n =* 32, 43%). For example, one respondent reported that they “would consider using ChatGPT in my future classroom to help with lesson plan ideas, strategies for behavioral issues, class projects, science experiments, etc.” Another respondent described how they would implement ChatGPT in the future, noting how this approach aligned with potential ethical concerns, writing:

In the future, I would like to use ChatGPT to plan fun activities for lessons for all many different subjects. I do not see it as cheating, because it is something that would benefit the students instead of hurting them. Getting ideas from ChatGPT does not take away the fact that I would still teach the students and interact with them in a meaningful way.

Finally, one respondent connected their plans for future use to their current classroom experiences, writing:

I plan to use [ChatGPT] to help me with lesson planning because of the lack of resources online. Personally, the last school I taught at did not provide a curriculum so teachers were forced to scramble for resources. AI was very helpful in getting me started with lesson planning.

#### Future use three: need knowledge for effective use

The tertiary emergent theme around future use of GenAI tools like ChatGPT for teachers was the importance of receiving proper professional development and training to use these tools effectively and be critical evaluators (*n =* 22, 30%). For example, one participant acknowledged their current limitations writing, “I would not feel comfortable doing that unless I learned more about it and how to teach students how to properly use it.” Another echoed this sentiment, reporting “I would like to use ChatGPT in my future classroom but would like to learn how to use it correctly before I implement it in my lessons or classroom activities.” Of note for this theme, 18 participants with prior experience noted the importance of expanding their knowledge and training to be able to use ChatGPT effectively in their future classroom, while only four participants without prior experience reported this theme.

## Discussion and significance

Based on the results above, this study suggests several contributions for both practice and future scholarship. Importantly, this study expands on existing scholarship by exploring preservice teachers’ perceptions on both future teachers’ and students’ use of AI as well as their intentions for use. These contributions are explored in detail below.

### Alignment across perceived affordances and limitations for teachers and students

As showcased above in Table 4, there was significant alignment across the emergent themes for perceived affordances and limitations for both teachers and students. Importantly, these perceived affordances also aligned with participants’ reported ideas for future classroom usage of GenAI tools like ChatGPT as well. Overall, the three emergent themes for affordances for students (Idea Generation - 58%, Knowledge for Effective Use - 19%, and Editing and Proofing - 11%) were nearly identical to the three emergent themes for affordances for teachers (Idea Generation - 42%, Lesson Planning - 37%, Knowledge for Effective Use - 18%). The exception being that “Lesson Planning” for teachers replaced the student theme of “Proofing,” although in practice, these two themes are likely similar. The emergent themes for limitations were also similarly aligned, with student themes (Become Dependent - 47%, Plagiarism - 45%, and Bias and Misinformation - 39%) being identical to the teacher themes (Bias and Misinformation - 51%, Become Dependent - 45%, Plagiarism and Job Risks - 10%). Interestingly, this clear alignment suggests that there may be broad humanistic concerns for GenAI usage that extend beyond the role of teacher or student (e.g., Sharma, 2024).

Other research has highlighted similar findings from preservice teachers (e.g., Bae et al., 2024). For example, Bae et al. (2024) found that preservice teachers in their study considered lesson planning, as well as brainstorming and idea generation, as potential affordances. Additionally, Bae et al. (2024) noted that while exposure to and experience with ChatGPT increased preservice teachers’ awareness and foundational knowledge, they still reported lingering uncertainty over adopting these tools in their classrooms. In other words, the strong alignment across perceived affordances and limitations for teachers and students suggests that preservice teachers do not view ChatGPT as a neutral classroom add-on tool, but rather as something that has the potential to reshape instructional and pedagogical practices. Recent research similarly shows that preservice teachers tend to recognize both the practical value of generative AI, but also the need for caution and additional training (e.g., Bae et al., 2024; Guan et al., 2025). Future work is needed to explore preservice teacher perceptions as they receive more advanced and in-depth training and experience with GenAI tools, to see if these findings remain consistent or shift as expertise deepens.

One of the most striking findings from this study was the asymmetry between preservice teachers’ intentions to use ChatGPT themselves (91%) and their willingness to support student use (19%). This gap suggests participants may be positioning ChatGPT as more of a professional aid for planning and preparation, while resisting its use as a learning tool for students. This positioning echoes recent literature showing growing awareness of GenAI’s usefulness paired with persistent uncertainty about classroom adoption (Bae et al., 2024; Guan et al., 2025). When unchallenged, this asymmetry could result in future classroom practices where teachers rely on AI for their own work needs while prohibiting or restricting student use, leading to inconsistent norms about acceptable use and assistance. Future research could explore preservice teacher professional development for supporting the creation of classroom norms, policies, and practices for AI use that do not require either unrestricted adoption or complete prohibition.

### ChatGPT and GenAI for supporting accessibility

While not a major emergent theme, six respondents (8%) discussed the possibility of using GenAI tools like ChatGPT to provide support for students with disabilities. For example, one student wrote: “I would use it to help me make accommodations for students that might be struggling.” Others discussed using ChatGPT to help with differentiating course materials or assisting with making materials more accessible to students with a variety of different learning needs. Research on supporting students with disabilities and special needs through GenAI tools like ChatGPT is an emerging area of research (e.g., Oh-Young and Karlin, 2025), but one that shows potential for future exploration. Importantly, supporting students with disabilities through GenAI often includes the disclosure of personal student information to the GenAI system, an idea that is in tension with what multiple participants shared in the results, and emphasizes the importance of exploring critical perspectives of GenAI within teacher education.

Although accessibility was not a dominant theme in participants’ responses, the fact that some preservice teachers identified ChatGPT and related GenAI tools as supports for students with disabilities is still important. This identification aligns with developing research around the use of GenAI as a possible aid for inclusion, differentiation, and Universal Design for Learning (UDL). For example, Evmenova et al. (2024) argue that GenAI can support English learners and students with disabilities by helping teachers create more flexible representations, scaffolds, and response options, while Song et al. (2024) similarly call for inclusive AI learning design grounded in UDL principles. Emerging empirical work also suggests that students with disabilities are already using generative AI to support reading, writing, self-organization, and academic work, while also reporting concerns about inaccuracy (Pierrès et al., 2025; Zhao et al., 2025).

However, the accessibility potential of ChatGPT must be weighed against concerns regarding privacy and disability-related data. If teachers use public-facing GenAI systems to tailor supports, accommodations, or individualized materials, they may inadvertently expose sensitive student information. Future research could explore best practices for professional development on supporting special education students while also being trained to use de-identified prompts, school-approved tools, human review processes, and more to ensure the safety of student data.

### Analysis through the EnCITE framework Lens

Viewed through the EnCITE framework, participants’ responses show that ChatGPT was not experienced as a simple tool to adopt or reject, but as a sociotechnical practice that preservice teachers were learning about, with, through, and against (Vogel et al., 2024). Participants learned with and through ChatGPT when they described using it for idea generation, lesson planning, editing, and differentiation, all of which positioned the tool as a resource for extending instructional design and efficiency. At the same time, they learned about and against ChatGPT when they emphasized the need to verify outputs, write thoughtful prompts, and remain cautious of plagiarism, dependency, bias, and misinformation. This pattern reinforces the argument that digital tools should not be treated as neutral classroom add-ons, because participants consistently framed ChatGPT as something that could reshape instruction and expectations for student thinking (Krutka et al., 2022; Vogel et al., 2024).

EnCITE’s five problem-and-opportunity spaces further clarify the tensions present in these findings. The technological and pedagogical spaces are evident in participants’ interest in teacher-facing uses such as brainstorming and lesson planning, while the definitional/ideological and political spaces appear in their significant hesitation about student use, especially around cheating, overreliance, misinformation, and the possibility of harm being unevenly distributed across learners. The developmental space is visible in the repeated call for more preparation and training, where even participants who saw clear benefits often said they needed additional professional development before using ChatGPT responsibly. Analyzed through the EnCITE framework, these results suggest that preservice teachers are approaching GenAI with cautious pragmatism rather than simple acceptance or rejection, a stance that also aligns with emerging preservice teacher research on GenAI adoption (Bae et al., 2024; Guan et al., 2025). This tension between possibility and harm leads directly to the importance of keeping critical perspectives on GenAI at the center of teacher education.

### Importance of critical perspectives of GenAI in teacher education

Just as future teachers need to understand the potential for emerging GenAI tools, they also need a critical understanding of how students’ data might be used for nefarious purposes (Yu and Guo, 2023), how biases and hallucinations can lead to misinformation and harmful information being shared (Banh and Strobel, 2023), the increasing environmental impact that running GenAI tools is having (Berthelot et al., 2024), and other problematic issues around this new technology. As the results from this study suggest, numerous participants reported on critical issues being a concern across their survey responses. More research is needed to explore the tensions that exist between these beneficial and critical perspectives of GenAI within teacher education.

The results also reinforce that critical perspectives on GenAI should be treated as a central component of teacher education rather than an optional activity. Recent scholarship has argued that AI literacy is increasingly discussed in education but remains underdeveloped in teacher education, particularly with respect to the practical and ethical knowledge teachers need for classroom decision-making (Sperling et al., 2024). This point is especially relevant in the current study given participants’ concerns about bias, hallucinations, dependence, and data use which suggests that preservice teachers are already noticing the ethical issues of these tools, even when they remain interested in using them. Recent research with preservice teachers indicates that many use AI only when warranted and still need stronger grounding in AI fundamentals and ethics before integrating it confidently into educational settings (Guan et al., 2025).

One potential extension of this work would be to consider how preservice teachers might translate these concerns into concrete classroom practices, especially around student data and privacy. For example, UNESCO (2023) guidance stresses that educational uses of generative AI should be human-centered and should protect data privacy. In practical terms, future teachers could address ChatGPT-related concerns by establishing clear classroom norms that prohibit entering personally identifiable, disability-related, behavioral, or assessment-related student information into public GenAI systems. Yan and Liu’s (2025) ethical framework for AI education further suggests the value of structured ethical review processes before adopting AI tools, which could help preservice teachers ask whether a tool is appropriate for a specific task, whose interests it serves, what risks it introduces, and what safeguards are necessary. Embedding this kind of ethical reasoning into teacher preparation would strengthen preservice teachers’ ability to support innovation while still protecting learners from harms related to surveillance, bias, and misinformation.

As mentioned above, the overall pattern in these findings could be characterized as cautious pragmatism about GenAI adoption rather than wholehearted optimism. As Königs (2022) describes, techno-optimism relates to broadly favorable expectations about a technology’s likely impact. In contrast, participants in this study described ChatGPT as useful for brainstorming, lesson planning, and efficiency while also identifying substantial risks related to dependency, plagiarism, bias, and misinformation. Rather than expressing confidence that GenAI will straightforwardly improve teaching and learning, these responses suggest that preservice teachers view ChatGPT as a tool whose value depends on context, professional judgment, and careful constraints. As described above, this interpretation is more consistent with recent research showing that preservice teachers often recognize the utility of GenAI while still expressing uncertainty about classroom adoption and a need for stronger AI literacy and ethical guidance (Bae et al., 2024; Guan et al., 2025). Teacher education, therefore, should prepare preservice teachers not only to use GenAI tools, but also to verify outputs, build critical literacy, set boundaries for appropriate use, and make principled decisions about when AI supports learning and when it may undermine it. Overall, these findings suggest that the central task for teacher education is not simply to encourage GenAI adoption, but to cultivate the critical, ethical, and pedagogical judgment needed to engage these tools responsibly.

## Conclusion

As GenAI tools like ChatGPT continue to become incorporated and integrated into everyday life, it is necessary for future teachers to have a fundamental understanding of what these tools can do, how they function, and their potential for supporting teaching and learning (Ottenbreit-Leftwich et al., 2023). As discussed by participants, GenAI tools have the potential to support teaching and learning in a myriad of ways, including supporting brainstorming, lesson planning, activity development, and differentiation processes (e.g., Baidoo-Anu and Ansah, 2023). However, these affordances do not come without limitations, risks, and the potential for harm, which must be actively considered and addressed when integrating these tools in teacher education. Beyond identifying affordances and limitations, this study shows that preservice teachers are already negotiating complex questions about learning, trust, and professional responsibility as they make sense of GenAI.

In addition to our study’s immediate findings, this work contributes to a growing but still emergent body of research on preservice teachers’ engagement with GenAI. By centering diverse preservice teacher voices across two institutional contexts and grounding experiences in interactive modules that structured participants’ engagement with ChatGPT, this study offers a distinctive contribution to the literature. It highlights both the promise of GenAI for lesson planning and idea generation, alongside immediate concerns related to dependency, bias, misinformation, and privacy. This work extends recent scholarship that emphasizes the importance of preparing future teachers not only with technical GenAI competencies but also with critical understandings of how to evaluate, question, and constrain AI in educational settings (Kasneci et al., 2023; Vogel et al., 2024). For teacher education programs, these findings suggest the need for required, scaffolded opportunities to engage with GenAI across coursework, including prompt design, output verification, hallucination detection, bias analysis, privacy-protective practices, and the development of clear classroom norms for both teacher and student use. Programs should also model how GenAI can be used for pedagogical tasks such as brainstorming, lesson-plan drafting, and differentiation, while still requiring human review and local contextualization.

Future research should examine how these perceptions and intentions develop over time and whether they translate into actual practice during student teaching and early-career teaching. Longitudinal studies could track whether the strong gap found here between willingness to use ChatGPT as a teacher and willingness to support student use persists, narrows, or widens with experience. Intervention studies could also test which teacher education approaches best strengthen candidates’ technical AI literacy, ethical reasoning, and ability to develop context-specific classroom policies. Additional work across content areas, institution types, and community contexts is needed, especially studies that examine multilingual learners, students with disabilities, and schools operating under different AI policies. Overall, this study demonstrates that preservice teachers’ beliefs about GenAI are marked by cautious pragmatism rather than straightforward acceptance or rejection. As GenAI tools continue to proliferate in teaching and learning spaces, the field must treat technical skill, critical inquiry, and justice-oriented judgment as inseparable components of teacher preparation.

## Data Availability

The raw data supporting the conclusions of this article will be made available by the authors, without undue reservation.
