# Supplementary material for: Balancing beliefs: exploring preservice teachers’ generative artificial intelligence perceptions and intentions for practice
Source: Front Artif Intell. 2026 Jun 1;9:1834667. doi: 10.3389/frai.2026.1834667 (PMC13265551; doi:10.3389/frai.2026.1834667)
Supplement: Supplementary file 1 [file Supplementary_file_1.docx]

**Appendix A**

*Module 3 Prompts, Primary ChatGPT Engagement*

For Prompt 1 you will be using the following prompt. Please remember to replace the underlined words with your own real-life experiences. More details in the video tutorial. ***(1) What was it like to grow up as a kid in (PLACE) during (TIME)?***

1. Please copy and paste your specific Prompt 1 ***Prompt***from ChatGPT

2. Please copy and paste your specific Prompt 1 ***Response*** from ChatGPT

3. Reflection 1.1 Tell us which parts of ChatGPTs Prompt 1 ***response*** were an accurate reflection of your own experiences? What parts did *not* describe your own experiences?

4. Reflection 1.2 What did you notice or wonder about from ChatGPT’s Prompt 1 ***response***?

For Prompt 2 you will be using the following prompt. Please remember to replace the underlined words with your own real-life experiences. More details in the video tutorial. ***(2) What was it like to grow up as a (IDENTITY) kid in (PLACE) during (TIME)?***

5. Please copy and paste your specific Prompt 2 ***Prompt***from ChatGPT

6. Please copy and paste your specific Prompt 2 ***Response*** from ChatGPT

7. Reflection 2.1 Tell us which parts of ChatGPTs Prompt 2 ***response*** were an accurate reflection of your own experiences? What parts did *not* describe your own experiences?

8. Reflection 2.1 What did you notice or wonder about from ChatGPT’s Prompt 2 ***response***?

For Prompt 3 you will be using the following prompt. Please remember to replace the underlined words with your own real-life experiences. More details in the video tutorial. ***(3) What cultural knowledge did families possess in (PLACE) during (TIME)?***

9. Please copy and paste your specific Prompt 3 ***Prompt***from ChatGPT

10. Please copy and paste your specific Prompt 3 ***Response*** from ChatGPT

11. Reflection 3.1 Tell us which parts of ChatGPTs Prompt 3 ***response*** were an accurate reflection of your own experiences? What parts did *not* describe your own experiences?

12. Reflection 3.2 What did you notice or wonder about from ChatGPT’s Prompt 3 ***response***?

For Prompt 4 you will be using the following prompt. Please remember to replace the underlined words with your own real-life experiences. More details in the video tutorial. ***(4) What cultural knowledge did (IDENTITY) families possess in (PLACE) during (TIME)?***

13. Please copy and paste your specific Prompt 4 ***Prompt***from ChatGPT

14. Response Please copy and paste your specific Prompt 4 ***Response*** from ChatGPT

15. Reflection 4.1 Tell us which parts of ChatGPTs Prompt 4 ***response*** were an accurate reflection of your own experiences? What parts did *not* describe your own experiences?

16. Reflection 4.2 What did you notice or wonder about from ChatGPT’s Prompt 4 ***response***?

For Prompt 5 you will be using the following prompt. Please remember to replace the underlined words with your own real-life experiences. More details in the video tutorial. ***(5) What was the history of (PLACE) during (TIME)?***

17. Please copy and paste your specific Prompt 5 ***Prompt***from ChatGPT

18. Please copy and paste your specific Prompt 5 ***Response*** from ChatGPT

19. Reflection 5.1 Tell us which parts of ChatGPTs Prompt 5 ***response*** were an accurate reflection of your own experiences? What parts did *not* describe your own experiences?

20. Reflection 5.2 What did you notice or wonder about from ChatGPT’s Prompt 5 ***response***?

For Prompt 6 you will be using the following prompt. Please remember to replace the underlined words with your own real-life experiences. More details in the video tutorial. ***(6) What was the history of (IDENTITY) from (PLACE) during (TIME)?***

21. Please copy and paste your specific Prompt 6 ***Prompt***from ChatGPT

22. Response Please copy and paste your specific Prompt 6 ***Response*** from ChatGPT

23. Reflection 6.1 Tell us which parts of ChatGPTs Prompt 6 ***response*** were an accurate reflection of your own experiences? What parts did *not* describe your own experiences?

24. Reflection 6.2 What did you notice or wonder about from ChatGPT’s Prompt 6 ***response***?

For Prompt 7 you will be using the following prompt. ***(7) Create your own prompt related to your own experiences around place, identity, language, education, history, and/or cultural knowledge.***

25. Please copy and paste your specific Prompt 7 ***Prompt***from ChatGPT

26. Please copy and paste your specific Prompt 7 ***Response*** from ChatGPT

27. Reflection 7.1 Tell us which parts of ChatGPTs Prompt 7 ***response*** were an accurate reflection of your own experiences? What parts did *not* describe your own experiences?

28. Reflection 7.2 What did you notice or wonder about from ChatGPT’s Prompt 7 ***response***?

**Appendix B**

*Module 4 Survey Instrument*


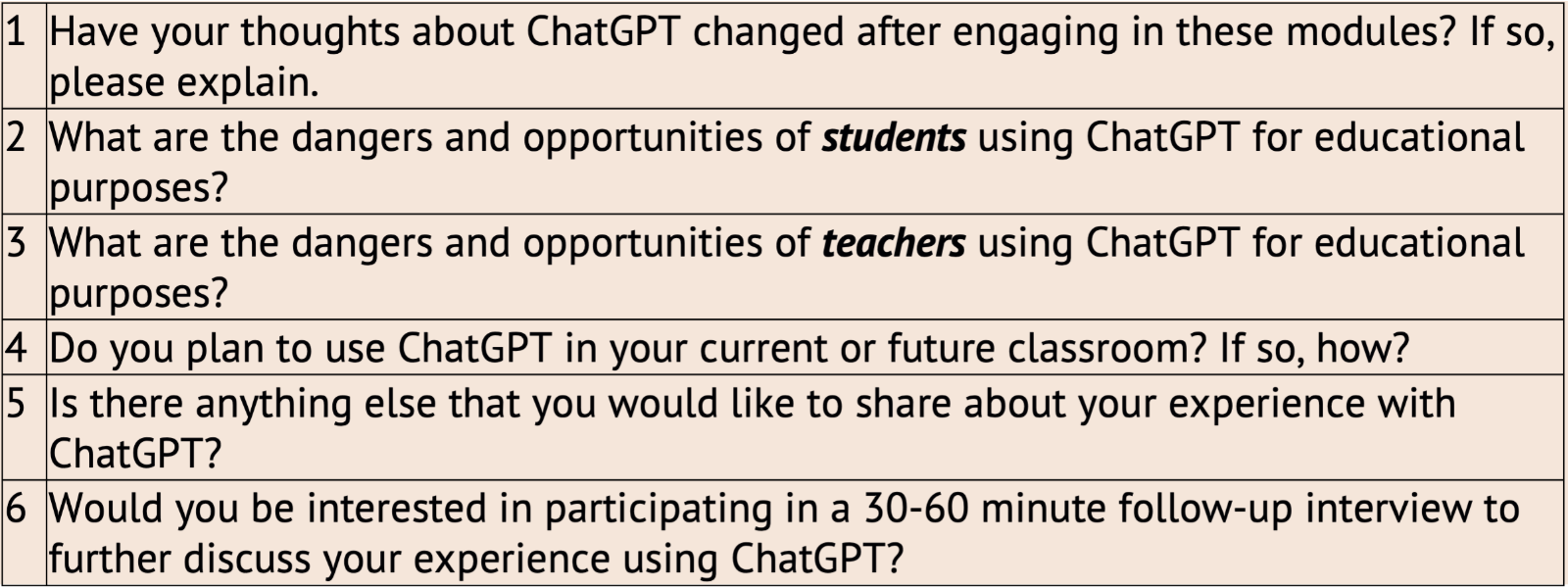


**Appendix C**

*Final Coding Schemes*

RQ1 - Student Affordances and Limitations Codes

| Student Limitation - Dependent |
| --- |
| Student Limitation - Misinfo |
| Student Limitation - Plagiarism |
| Student Limitation - Unsafe |
| Student Affordance - Exploration |
| Student Affordance - Proofing |
| Student Affordance - Know How To Use |
| Student Affordance - Accessibility |

RQ2 - Teacher Affordances and Limitations Codes

| Teacher Limitation - Dependent |
| --- |
| Teacher Limitation - Misinfo |
| Teacher Limitation - Job Risk |
| Teacher Limitation - Plagiarism |
| Teacher Limitation - None |
| Teacher Affordance - Exploration |
| Teacher Affordance - Lesson Planning |
| Teacher Affordance - Save Time |
| Teacher Affordance - Accessibility |
| Teacher Affordance - Know How To Use |
| Teacher Affordance - Improved Learning |
| Teacher Affordance - None |

RQ3 - Teacher and Student Future Use Codes

| Teacher Use - Lesson Planning |
| --- |
| Teacher Use - Exploration |
| Teacher Use - Accessibility |
| Teacher Use - Basic Tasks Save Time |
| Teacher Use - Know How To Use |
| Teacher Use - No |
| Student Use - Exploration |
| Student Use - Know How To Use |
